# Prevalence of Stroke and Associated Risk Factors in Sleman District of Yogyakarta Special Region, Indonesia

**DOI:** 10.1155/2019/2642458

**Published:** 2019-05-02

**Authors:** Ismail Setyopranoto, Halwan Fuad Bayuangga, Andre Stefanus Panggabean, Sarastiti Alifaningdyah, Lutfan Lazuardi, Fatwa Sari Tetra Dewi, Rusdy Ghazali Malueka

**Affiliations:** ^1^Neurology Department, Faculty of Medicine, Public Health and Nursing, Universitas Gadjah Mada, Yogyakarta, Indonesia; ^2^Sleman Health and Demographic Surveillance System (HDSS), Universitas Gadjah Mada, Yogyakarta, Indonesia; ^3^Department of Health Policy and Management, Faculty of Medicine, Public Health and Nursing, Universitas Gadjah Mada, Yogyakarta, Indonesia; ^4^Department of Health Behaviour, Environment and Social Medicine, Faculty of Medicine, Public Health and Nursing, Universitas Gadjah Mada, Yogyakarta, Indonesia

## Abstract

**Background:**

Stroke remains one of the most common noncommunicable diseases among Indonesian populations. This study aimed to identify the prevalence of stroke and its associated risk factors in the Sleman District of Yogyakarta Special Region, Indonesia.

**Method:**

This study was a secondary analysis of community-based data collected by the Sleman Health and Demographic Surveillance System (HDSS) in 2016. Basic demographic and socioeconomic data were collected. Additional questions about history of stroke and other chronic diseases were interviewed as a self-reported diagnosis. History of hormonal contraceptives use and dietary patterns were also collected. We examined the association between the prevalence of stroke and risk factors, namely, age, gender, self-reported history of chronic diseases, hormonal contraceptives use, and high-risk dietary patterns.

**Results:**

The survey included 4,996 households composed of 20,465 individuals. Data regarding stroke incidents were available from 13,605 subjects aged ≥20 years old. Among them, a total of 4,884 subjects also have data regarding stroke risk factors. The overall prevalence of stroke in Sleman District was 1.4% (0.5% men and 0.90% women). The prevalence increased with additional decades of age (*p*<0.001). In a multivariable model, increasing age, self-reported history of hypertension (OR=8.37, 95%CI: 4.76 to 14.69), and self-reported history of diabetes mellitus (OR=2.87, 95%CI: 1.54 to 5.35) were significantly associated with stroke.

**Conclusions:**

A community-based survey in Indonesia showed a high prevalence of stroke which was associated with increasing age, hypertension, and diabetes mellitus. These findings suggest that preventive actions against the aforementioned modifiable risk factors should be prioritized.

## 1. Introduction

Noncommunicable diseases (NCDs) are a major health problem globally with 63% of deaths due to NCDs, and it is projected to increase by 15% between 2010 and 2020 [1, 2]. The predominant NCDs reported were cardiovascular diseases, diabetes, and chronic respiratory diseases [2]. Of these, stroke is the second leading cause of death after ischemic heart disease and the most common reason of acquired disability in adults worldwide [3, 4]. The burden of NCDs including stroke has remained stable in high-income countries over the past decade, while the number is rising to almost 50% of total disease burden in low-income and middle-income countries [5]. This changing trend can be attributed to population aging and the increase of modifiable risk factors for cardiovascular diseases [5, 6].

Indonesia has a high burden of stroke, and it became the number one cause of death, killing 328.5 thousand people (21.2% of total deaths) in 2012 according to WHO [7]. Among countries in Asia, Indonesia has the highest stroke mortality rate with age-sex standardized mortality in 2010 at 193.3/100,000 person-years [8]. The Indonesian Basic Health Research revealed that the prevalence of stroke in Indonesia is 12.1 per mil with the highest number in North Sulawesi province (17.9%) followed by Yogyakarta province (16.9%) [9]. It was also reported that the prevalence of stroke is increasing as people get older with the peak age at 75 or older. The stroke prevalence is the same in both sexes [5]. Worldwide, stroke is more common among men, but women are more severely ill [10].

The best method to control the stroke burden and to meet the global goal of 2% reduction each year in stroke death rates is by primary prevention via early detection of stroke risk factors [5]. Risk factors for stroke are usually divided into nonmodifiable (age, sex, ethnicity, low weight at birth, and inherited diseases) and modifiable risk factors (hypertension, diabetes mellitus, heart diseases, smoking, dyslipidemia, obesity, metabolic syndrome, use of oral contraceptive drugs, and others) [11]. A study showed that 90.5% of the global burden of stroke was attributable to the modifiable risk factors, including 74.2% of them related to lifestyle (i.e., smoking, poor diet, and low physical activity) [12]. However, in Indonesia, studies that specifically look at the prevalence of stroke and the risk factors associated with it are still lacking.

To develop early prevention of stroke by controlling risk factors, a survey about the characteristics of risk factors in stroke patients in the community needs to be managed in a structured, systematic, and integrated manner in a regional network. In this study, we use the secondary data from Sleman Health Demographic and Surveillance System (HDSS) which was conducted by the Universitas Gadjah Mada as a longitudinal surveillance focusing on NCDs, communicable disease, and healthcare facilities [13]. This study was conducted to determine the characteristics of stroke risk factors in stroke patients in Sleman District of Yogyakarta Special Region, Indonesia.

## 2. Methods

The Sleman Health Demographic and Surveillance System (HDSS) is a longitudinal and community-based survey design established in 2014 through a close collaboration between the Faculty of Medicine, Public Health and Nursing, Universitas Gadjah Mada, and the Government of Sleman District, Yogyakarta Special Region, Indonesia [13]. The main purpose was to provide a dynamic cohort of demographic, socioeconomic, and health transitions of the population in the area [13]. Thus, it complemented existing local and national censuses in supporting evidence-based policy making.

The sample size was calculated as described previously [13]. Based on the calculation, the minimum sample size was 4942 households. For the baseline survey in 2015, the subjects included people who had been living in Sleman for at least six months before the sample verification period and it excluded those who refused to participate, were severely ill, and/or were unable to communicate [13]. A two-stage cluster sampling with probability proportional to size approach was applied to define the sampling frame. First, 216 Census Blocks (CBs) were randomly selected from 3,513 CBs, with 184 CBs from urban area and 32 CBs from rural area. Next, using systematic random sampling, 25 households were selected from each CB for a total of 5,400 households [13]. Two hundred forty-seven households were failed to be interviewed resulting in 5,147 households that participated in the baseline survey in 2015. In the baseline, demographic data including census data, vital demographic events in the last year, socioeconomic data, and verbal autopsy for death event were collected. In 2016, the second cycle included 4,996 households composed of 20,465 individuals (97.0% response rate) [13]. One hundred fifty-one households from the baseline survey were not interviewed in the second cycle because of exclusion criteria (moving to another place, death, refused to be interviewed, etc.) [13]. In this year the survey updated already collected data and introduced new variables, namely, frequencies of disease morbidity and associated risk factors, as well as community's access to health services. The same sampling frame was maintained throughout repeated cycles of data collection after considering every birth and death and migration events [13]. Therefore, this design provided a valuable platform for cohort studies and cross-sectional analysis.

Trained enumerators gathered relevant data from respondents using the Computer-Assisted Personal Interview (CAPI) system during home visits. Standardized questionnaires developed by the INDEPTH Network that had been applied in other HDSS in Indonesia were used to capture demographic and socioeconomic data [13, 14]. Additional structured precoded questions about history of stroke and other chronic diseases were interviewed as self-reported diagnosis. History of hormonal contraceptives use and risk factors related to dietary patterns were also asked. For the purpose of this current study, we confined our analysis to the community-based prevalence of stroke and its associated risk factors from 2016 data. We also limited our samples to respondents aged ≥20 years because stroke is mostly found after this age. Furthermore, a large percentage of respondents were unable to report their typical dietary patterns (35.9%). As a result, the sample included 4,884 respondents with complete data for each variable.

To ease the extrapolation of study results, data were adjusted to age-groups and gender of Sleman population according to the Indonesian Central Bureau of Statistics (*Badan Pusat Statistik/BPS*) survey in 2015 [15]. Descriptive analysis was performed to identify characteristics of stroke cases and non-cases groups. Numerical data are presented as mean and were interpreted using binomial logistic regression analysis. Meanwhile, categorical variables are presented as proportions and were analyzed using the Chi-square test. At last, variables were entered into multivariable logistic regression analyses to determine independent associations of each variable with the prevalence of stroke. This study has received ethical approval from the Institutional Review Board, Faculty of Medicine, Public Health and Nursing, Universitas Gadjah Mada, Yogyakarta, with the number KE/FK/0434/EC. Ethical approval will be updated annually. This manuscript was written in accordance with cross-sectional reporting principle, using Strengthening the Reporting of Observational Studies in Epidemiology (STROBE) guideline [16].

## 3. Results

Data regarding stroke incidence were available from 13,605 subjects aged ≥ 20 years old. Among them, a total of 4,884 subjects also have data regarding stroke risk factors. From the 13,605 subjects with stroke data, 197 (1.4%) of them had stroke. From the 4,884 subjects with data of stroke and risk factors, 69 of them had stroke (1.4%). For further analysis, only subjects with complete data about the stroke risk factors were included. Descriptive characteristics of the sample are reported in Table 1. The mean age was 49.91 (±14.16) years old, and there was a significant difference in means between cases and non-cases group (p<0.001). Sixty-four percent of the samples were females, and 44 of them had stroke cases (1.41%). However, when compared to male populations (prevalence = 1.42%), there were no statistical differences between groups.

History of hypertension was found in 18.37% subjects, and 5.68% of them had stroke. In the stroke group, 73.91% of them had hypertension history. When compared with the non-stroke group, there was a significant association between hypertension and stroke (*p*<0.001). Diabetes mellitus was also found in 4.95% of individuals, and 6.61% of them had stroke. There was also a significant association between diabetes mellitus and stroke (*p*<0.001). Four from 69 (5.79%) individuals in the stroke group had history of cardiovascular disease, and this was significantly higher than in the non-stroke group (p = 0.001). Hormonal contraception users were found in 24.47% of female populations, but there was only one stroke case from 766 users (*p*=0.001).

High-risk diet was analyzed as a numeric variable with intake per day as its unit. The mean intake of high-salt diet was almost one intake/day (0.95 ± 1.13) and there was no significant difference of mean intake between the stroke and non-stroke groups (*p*=0.290). These insignificant differences were also found in high-calorie (*p*=0.900) and high-fat diet (*p*=0.778). For processed food consumption, the mean intake was 0.07 intakes per day, which meant there was only one intake every 15 days. There was a significant difference (*p*<0.001) between the stroke group (0.03 ± 0.13) and the non-stroke group (0.06 ± 0.26). The mean intake for salted fish consumption was 0.11 intakes per day, and the stroke group had a significantly higher intake (0.13 ±0.37) than the non-stroke group (0.11 ± 0.26,* p*=0.008). Inversely, the mean intake for instant noodles consumption was higher in the non-stroke group (0.17 ± 0.29) than the stroke group (0.11 ± 0.17,* p*=0.004).

The number of cases divided by age and sex was reported in Table 2. The highest prevalence of stroke was in ≥70-year-old group (31.88%). For the female group, the highest prevalence was between 50 and 59 years old (15 cases). On the other side, for the male population, the highest was in ≥70 years old (8 cases). Between each of the age groups, there were no significant differences between the male and female groups in stroke prevalence (*p*>0.05). To extrapolate this study in a true population, we adjusted the prevalence rate of stroke in each age group and sex (Table 3). The highest prevalence rate of stroke was between 50 and 59 years old, with a prevalence rate of 174.12. For the male population, the highest was also between 50 and 59 years old (prevalence rate=129.46). For the female population, the age ≥70 years (203.43) was slightly higher than the 50–59-year old group (202.25).

To determine the true risk factors that associate with stroke cases, all the independent variables (gender, age group, history of hypertension, history of diabetes mellitus, history of cardiovascular disease, and high-risk dietary patterns) were analyzed with multivariable analysis using binomial logistic regression statistical method (Table 4). History of hormonal contraceptives use was not included in the analysis because this data was only collected from female subjects. From all of the variables that entered the logistic regression, only age (*p*<0.001), hypertension (*p*<0.001), and diabetes mellitus (*p*=0.001) were associated significantly and independently with stroke prevalence. For age variables, every increase of one decade associated with higher number of stroke cases. Individuals with history of hypertension had 8.37 times higher risk of having stroke than in the normal population. Subjects with history of diabetes mellitus had 2.87 odd ratios for having stroke. At last, from all of these three variables, the history of hypertension was the most important risk factor for the Sleman population. Area under Receiver Operating Characteristic (ROC) curve for this binomial logistic regression model is 0.87, indicating excellent discrimination (graph not shown) [17].

## 4. Discussions

This study demonstrated that the stroke prevalence in Sleman population was 14 per thousand, higher than Indonesia prevalence number which was 12.1 per thousand [9]. Yet, this number was still low compared to Yogyakarta Special Region's prevalence, which was 16.9 per thousand [9]. This high prevalence could be explained by Yogyakarta Special Region life expectancy number which was the highest in Indonesia with 73.62 [15].

The stroke prevalence from this study was still low compared to the global prevalence of 27 per thousand and USA prevalence of 26 per thousand in those over 20 years in 2014 [18]. Among countries in Asia, the number was also lower than the findings from Korea (15.9 per thousand) and Thailand (18.5 per thousand), but higher than the findings from China (2.6-7.19 per thousand) and India (5.45 per thousand) [19–22].

Indonesia stroke prevalence tended to increase since the data from the Ministry of Health showed that stroke prevalence in 2007 was 8.3 per thousand which increased to 12.1 per thousand in 2013 [10, 23]. The increasing trend of stroke was similar in most Asian countries due to the aging of the population and unhealthy lifestyle [24]. Another possible cause for the increased prevalence that we need to consider is the decrease in mortality rate for stroke in Indonesia. However, this is unlikely to be the cause in Indonesian population since previous studies showed an increase in age-sex standardized mortality/100,000 person-years from 99 in 2002 to 193.3 in 2010 [8, 25].

Stroke has been considered to be affected by nonmodifiable risk factors including gender and age. This study showed that stroke prevalence in females was slightly higher than in males but was not statistically significant. This finding was concordant with data from the American Heart Association that found that globally, in the years 2011-2014, the stroke prevalence in females was 2.6%, higher than in males (2.4%) [26]. Data in Indonesia also showed similar result, with stroke prevalence at 12.1% in females and 12% in males [9].

However, it was contrary to findings from other studies which said that stroke was more prevalent in males than females [27–29]. Recent studies showed that profiles of baseline risk factors and heritability of ischemic stroke are higher in females than in males [30]. Furthermore, the higher lifetime risk in women than men increases with the use of birth control pills and pregnancy [31]. Previous study showed that age-standardized mortality rate of stroke in Indonesia is equal between males and females (95 vs. 94 mortality/100,000 person-years, respectively) [32].

Age is one of the risk factors for chronic diseases, including cancer, cardiovascular diseases, and neurodegeneration [33]. For stroke, age is a continuous risk factor with a two-fold increase in the incidence and prevalence rates for each successive five years after age 65 [11]. In this study, the ratio from each age group was 1.73 which means almost two times increased risk of stroke for every ten years' addition. Another study also found that the incidence of stroke doubled for each decade after 55 years of age [34]. This finding could be explained by the increase in other risk factors such as type 2 diabetes, high blood pressure, and atherosclerosis as people get older.

Uncontrolled blood pressure is one of the most common risk factors for stroke, consistent with the finding in this study which showed that 73.9% of stroke subjects had hypertension history. The prevalence risk of hypertension in this study was the highest among other chronic diseases (diabetes and heart disease) with 8.37. In a cohort study from Sweden, the relative risk of hypertension was 2.55 in untreated hypertension patients and 4.30 in treated subjects with uncontrolled blood pressure [35]. Another study found that high blood pressure multiplies the risk for stroke as much as 4-fold [36]. Hypertension can cause development of atherosclerotic plaque, lipohyalinosis of penetrating artery, hypertrophy, remodeling of smooth muscle cell, reduction of cerebral blood flow, and arterial baroreflex dysfunction [37]. These mechanical changes can lead to either occlusion or degenerative change that causes blood vessels to be prone to rupture or bleeding [37]. Giving therapy for hypertension can avoid stroke complications. Therefore, it is important for physicians to monitor and control blood pressure in the normal range. However, it should be noted that previous studies have shown that hypertension is not a major risk factor for all ischemic stroke subtypes. Hypertension was the main cardiovascular risk factor only for lacunes and atherothrombotic infarction, that is, ischemic stroke associated with small- and large-artery disease [11]. In the future, it will be interesting to analyze the prevalence and risk factors for stroke in various ischemic stroke subtypes in this population.

Other chronic diseases such as diabetes and heart disease could increase the risk of stroke attack. In this study, subjects with diabetes and heart disease were 2.87 times and 2.02 times more susceptible to have stroke, respectively. Diabetes can cause pathologic changes in blood vessels at various locations and can lead to stroke if cerebral vessels are directly affected [38]. The data from the Greater Cincinnati/Northern Kentucky stroke study showed the increased risk of ischemic stroke in diabetes patients, and the risk was most striking before the age of 55 years in African Americans and before the age of 65 years in whites [39]. It strengthens the theory about the higher risk for stroke in young population with diabetes. Similar to diabetes, heart disease also contributes to the higher incidence of stroke. Several studies reported that one-third of stroke patients had heart diseases, with the most important risk factors being atrial fibrillation (AF) and atrial flutter [40, 41]. AF can cause stroke due to stasis of blood in the fibrillating left atrium triggering thrombus formation and embolization to the brain [11].

The use of hormonal contraceptive in women has been associated with an increased risk of stroke especially ischemic stroke, but the absolute risk remains very small [29, 42]. On the contrary, the finding from this study showed that hormonal use protected the subjects from stroke. One study explained that the risk of stroke associated with low-dose oral contraceptives (containing low doses of estrogens) in women without additional risk factors (*e.g., *cigarette smoking or history of thromboembolic episodes) appears low [11]. However, the association between hormonal contraception and stroke needs to be further investigated.

The incidence of stroke is also associated with dietary habit. Diets high in salt, fat, and sugar influence the risk of other diseases contributed to stroke, such as high blood pressure, dyslipidemia, and diabetes [43]. A higher sodium intake increases the risk of stroke, whereas a higher consumption of potassium was associated with a reduction in the risk of stroke [11]. A Mediterranean diet, or a diet high in fruits and vegetables, reduces the risk of stroke [43]. Our study, however, did not show independent association between dietary patterns and stroke. There could be a recall bias and measurement error during the study period.

Based on this study the most important risk factors for stroke in this population are hypertension and diabetes mellitus, especially in older patients. Strategy for stroke prevention in Sleman must include early detection and treatment of those conditions. It is important to control risk factors to reduce stroke burden, and it can be accomplished by two strategies: primary prevention and secondary prevention. The most cost-effective primary prevention is to quit smoking. Other primary preventions are having exercise at least 150 min/week, diet high in fiber, fruit, and vegetable intake and low in sugars and salt, managing weight, and pharmacotherapy to control hypertension and blood glucose [44]. Secondary prevention refers to the treatment of individuals who have already had a stroke, which may include the use of platelet anti-aggregation, antihypertensive, statins, and lifestyle interventions.

This study has several weaknesses. First, this study only covers one district area in the province of Yogyakarta, so that it may not be able to represent other regions with different characteristics. Second, there are many respondents who do not report on dietary patterns so that there is a risk of bias. Third, history of hypertension, diabetes, and heart disease was obtained through respondents' reports without cross-validation with hospital medical records. Fourth, this study does not take into account ischemic stroke subtypes. Fifth, data regarding the treatment of hypertension, diabetes, and heart disease was not collected. This data is very important as a part of the primary prevention of stroke. Sixth, several other risk factors such as smoking and physical activity have not been included. Further research needs to be done to address these limitations. Hospital-based research on stroke and the risk factors associated with it should also be carried out as a comparison with the results of this community-based study.

## 5. Conclusions

A community-based survey in Sleman District of Yogyakarta Special Region, Indonesia, showed a high prevalence of stroke which was associated with increasing age, hypertension, and diabetes mellitus. These findings suggest that preventive actions, especially against the aforementioned modifiable risk factors, should be prioritized.

## Figures and Tables

**Table 1:** Descriptive statistics of study samples stratified by case.

Characteristics	All (n=4884)	Stroke (n=69)	Non-stroke (n=4815)	*P* value
Age (years), mean (SD)	49.91 (±14.16)	63.10 (± 11.54)	49.72 (±14.11)	<0.001*∗*
Gender				
Male, n (%)	1754 (35.91%)	25 (36.23%^#^)	1729 (35.9%^#^)	0.956
Female, n (%)	3130 (64.09%)	44 (63.77%^#^)	3086 (64.1%^#^)	
Self-reported history of hypertension, n (%)	897 (18.37%)	51 (73.91%^#^)	846 (17.57%)	<0.001*∗*
Self-reported history of diabetes mellitus, n (%)	242 (4.95%)	16 (23.19%^#^)	226 (4.69%^#^)	<0.001*∗*
Self-reported history of cardiovascular diseases, n (%)	62 (1.27%)	4 (5.9%^#^)	58 (1.2%^#^)	0.001*∗*
Past and/or current use of hormonal contraceptives†, n (%)	766 (24.47%)	1 (1.4%^#^)	765 (15.89%^#^)	0.001*∗*
Oral, n (%)	180 (5.75%)	0 (0%^#^)	180 (3.7%^#^)	
Injectable, n (%)	557 (17.79%)	1 (1.45%^#^)	556 (11.55%^#^)	
Implant, n (%)	59 (1.88%)	0 (0%^#^)	59 (1.2%^#^)	
High-risk dietary pattern				
High-salt diet, mean intake per day (SD)	0.95 (± 1.13)	0.86 (± 1.08)	0.95 (± 1.13)	0.290
High-calorie diet, mean intake per day (SD)	1.57 (± 1.22)	1.65 (± 1.53)	1.57 (± 1.22)	0.900
High-fat diet, mean intake per day (SD)	1.13 (± 1.01)	1.17 (± 1.00)	1.13 (± 1.00)	0.778
Processed foods, mean intake per day (SD)	0.07 (± 0.26)	0.03 (± 0.13)	0.06 (± 0.26)	<0.001*∗*
Salted fish, mean intake per day (SD)	0.11 (± 0.26)	0.13 (±0.37)	0.11 (± 0.26)	0.008*∗*
Instant noodles, mean intake per day (SD)	0.17 (± 0.28)	0.11 (± 0.17)	0.17 (± 0.29)	0.004*∗*

*∗p* value <0.05 is considered significant.

†Data were collected from and calculated among females subjects only.

^#^Percentage was calculated as proportion of stroke or non-stroke sample having the risk factors.

**Table 2:** Age- and gender-specific prevalence of stroke.

Age groups (years)	Stroke cases, n (percentage; 95% CI)			*P* value*∗*
	Total (n=69)	Male (n=25)	Female (n=44)	
20-29	1 (0.30; 0.01-1.68)	0 (0)	1 (0.42; 0.01-2.35)	1.000
30-39	0 (0)	0 (0)	0 (0)	N/A
40-49	6 (0.47; 0.17-1.03)	4 (0.94; 0.26-2.39)	2 (0.24; 0.03-0.86)	0.102
50-59	21 (1.80; 1.12-2.74)	6 (1.34; 0.49-2.90)	15 (2.08; 1.17-3.41)	0.355
60-69	19 (2.61; 1.58-4.04)	7 (2.17; 0.88-4.41)	12 (2.95; 1.54-5.11)	0.507
≥70	22 (4.47; 2.82-6.69)	8 (3.33; 1.45-6.46)	14 (5.55; 3.07-9.15)	0.233

*∗p* value <0.05 is considered significant. Chi-square test was used for the comparison between males and females in each age group.

**Table 3:** Age- and gender-adjusted prevalence rate of stroke*∗*.

Age groups (years)	Stroke prevalence rate per 100,000 population (95% CI)		
	Total	Male	Female
20-29	50.02 (1.65-277.32)	0	70.06 (1.64-386.95)
30-39	0	0	0
40-49	63.94 (22.97-139.18)	126.75 (35.09-322.63)	32.13 (4.05-116.33)
50-59	174.12 (108.37-265.13)	129.46 (47.26-279.70)	202.25 (113.59-331.06)
60-69	136.83 (82.95-212.11)	112.10 (45.52-228.13)	157.48 (82.06-272.27)
≥ 70	143.88 (90.74-215.27)	92.60 (40.28-179.46)	203.43 (112.42-335.06)

*∗*Age- and gender-adjustments were based on the Indonesian Central Bureau of Statistics (*Badan Pusat Statistik/BPS*) survey in 2015.

**Table 4:** Multivariable logistic analysis of stroke risk factors among study samples.

Category	Subcategory	OR (95% CI)	*P* value
Gender	Female	1.00 (referent)	0.600
	Male	0.87 (0.51-1.47)	
Age group (years)	20-29	1.00 (referent)	<0.001*∗*
	30-39	-	
	40-49	0.91 (0.29 – 2.83)	
	50-59	1.10 (0.54-2.25)	
	60-69	1.25 (0.71-2.19)	
	≥70	1.41 (0.90-2.19)	
Self-reported history of hypertension	No	1.00 (referent)	<0.001*∗*
	Yes	8.37 (4.76-14.69)	
Self-reported history of diabetes mellitus	No	1.00 (referent)	0.001*∗*
	Yes	2.87 (1.54-5.35)	
Self-reported history of cardiovascular disease	No	1.00 (referent)	0.221
	Yes	2.02 (0.65-6.27)	
High-risk dietary patterns	High-salt diet	0.90 (0.71-1.15)	0.410
	High-calorie diet	1.11 (0.92-1.34)	0.239
	High-fat diet	1.11 (0.86-1.43)	0.436
	Processed foods	0.34 (0.28-4.09)	0.395
	Salted fish	1.81 (0.93-3.56)	0.083
	Instant noodles	0.66 (0.15-2.86)	0.583

Note: *∗p value *< 0.05 is considered significant.

## Data Availability

The data used to support the findings of this study are available from the corresponding author upon request and with permission of the Sleman Health and Demographic Surveillance System (HDSS), Faculty of Medicine, Public Health and Nursing, Universitas Gadjah Mada.
